# Use of a Double Reverse Traction Repositor *versus* a Traction Table for the Treatment of Intertrochanteric Femur Fractures: A Comparative Study

**DOI:** 10.1111/os.12956

**Published:** 2021-05-05

**Authors:** Mingming Yan, Letian Kuang, Jiangdong Ni, Muliang Ding, Junjie Wang, Jun Huang, Deye Song

**Affiliations:** ^1^ Department of Orthopaedic Surgery The Second Xiangya Hospital, Central South University Changsha China

**Keywords:** Double reverse traction repositor, PFNA‐II, Traction table, Unstable intertrochanteric fracture

## Abstract

**Objective:**

The aim of the present study was to compare the clinical results for unstable femoral intertrochanteric fractures treated with a double reverse traction repositor (DRTR) and those treated using a traction table with the Asia proximal femoral nail antirotation (PFNA‐II).

**Methods:**

A retrospective study was performed including 95 patients with AO/OTA type 31‐A2 and 31‐A3 unstable femoral intertrochanteric fractures who underwent DRTR or traction table‐facilitated PFNA‐II nailing from April 2015 to December 2018 in our traumatic center. Demographics, duration of operation, blood loss, part loading time after surgery, fracture healing time, and early and late complications were assessed. Clinical and radiological outcomes were collected to compare the differences between the two groups.

**Results:**

A total of 95 unstable intertrochanteric fracture patients treated with the PFNA‐II were analyzed. Of these cases, 56 patients were treated with a DRTR and the other 39 patients were treated using a traction table to achieve fracture reduction. No patients died during surgery and hospitalization. There were no significant differences in respect to demographics and fracture characteristics of cases enrolled. The total operative time was significantly longer in the traction table group than in the DRTR group (72.5 ± 6.1 min for the traction table and 63.0 ± 4.1 min for the DRTR group, *P* < 0.001). No significant differences were observed in intraoperative blood loss and duration of hospitalization. The periods of follow up ranged from 12 to 31 months among all patients. At the last follow up, the Harris hip score (HHS) in the DRTR group was excellent in 10 patients (17.9%), good in 36 (64.3%), fair in 8 (14.3%), and poor in 2 (3.6%). These scores were comparable to those in the traction table group, which were: excellent in 8 patients (20.5%), good in 24 (61.5%), fair in 6 (15.4%), and poor in 1 (2.6%). Regarding the radiological evaluation, excellent rates of reduction rate were achieved in 39 cases (69.6%) in the DRTR group, which was comparable to 19 cases (48.7%) in the traction table group. In addition, the mean fracture healing time after surgery was 20.6 ± 2.3 weeks in the DRTR group and 21.4 ± 3.4 weeks in the traction table group, which did not reach a significant difference (*P* = 0.18). During the follow up, 6 cases of thigh pain, 4 cases of deep vein thrombosis, and 1 case of fracture of the anterior superior iliac spine were reported in the DRTR group. In the traction table group, there were 2 cases of deep vein thrombosis and 3 cases of thigh pain.

**Conclusion:**

When using the PFNA‐II for unstable intertrochanteric fractures, the DRTR was superior to the traction table in respect to operative time and duration of patient position, despite an additional ipsilateral anterior superior iliac spine (ASIS) incision and drilling of the ASIS and the femur condyle.

## Graphical abstract

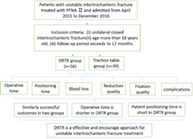

Use of a DRTR or a traction table achieves similarly successful results for unstable intertrochanteric fractures. Moreover, the DRTR is superior to the traction table with respect to operative time and duration of patient position.

## Introduction

In the elderly, hip fractures are associated with an approximate 25% mortality rate within the first year of fracture^1^. With the rapidly aging population and longer life expectancy, geriatric hip fractures are becoming a heavy burden to society and the healthcare system. An epidemiological study by Zhu *et al*. suggests that the prevalence of hip fractures will substantially increase in China, from 0.7 million cases in 2013 to 4.5 million cases in 2050^2^. This will have a heavy burden on the healthcare system and society. Although remarkable progress has been made during the past three decades, management of intertrochanteric fractures, especially unstable intertrochanteric fractures, remains a huge challenge for orthopaedic surgeons^3^. The ratio of intertrochanteric fracture treatment failure can be as high as 20%, even with the current techniques and the application of implants. Moreover, elderly people who experience trochanteric fractures are likely to have underlying conditions and comorbidities, making them more vulnerable to fracture‐associated complications and death than young patients. Therefore, a better understanding of the appropriate management of patients with intertrochanteric fractures is important^4^.

It has been well established that surgical treatment should be recommended for intertrochanteric fractures^4^. The primary goal of the operation is to obtain stable fixation to enable early mobilization and fracture union. The quality of the reduction, implant choice, and placement are also determinative factors influencing the intertrochanteric fracture outcomes^5^. Dynamic hip screws, femoral trochanter stabilization plates, proximal femoral plates, intramedullary nails, and hip prostheses are the preferred implants for intertrochanteric fracture fixation. After first being described in the 1980s, the intramedullary nail has become the first choice for unstable trochanter fractures due to its advantages in terms of biomechanics, minimal soft tissue injury, and few complications^6^. To obtain a satisfactory outcome, the reduction should be achieved prior to the insertion of the intramedullary nail; otherwise, failure of the surgical intervention will be inevitable. As an effective traction tool, the traction table has been widely used to facilitate the reduction of fractures around the hip. However, there are still several limitations in the application of the traction table. First, as a traction table provides traction force through traction between the foot and perineal post, the force will be attenuated over the knee and ankle joint. Second, a great variety of complications associated with traction table usage have been reported, including iatrogenic injuries, soft tissue contusions, compartment syndrome, crush syndrome, and vascular injuries^7^. These complications can have devastating consequences for the patient, which significantly compromises the application of a traction table. Moreover, it is difficult to establish a nail entry point to the greater trochanter in obese patients. To avoid these potential disadvantages, the double reverse traction repositor (DRTR) has emerged as an alternative to the traction table for the closed reduction of fragments and proximal femoral nail antirotation (PFNA)‐II fixation.

The DRTR was designed by Zhang and his colleagues to achieve a closed reduction of a displaced fracture based on a concept of skeletal distraction. Zhang *et al*. reported that patients with closed formal shaft fractures who were treated with a DRTR had a lower open reduction and nonunion rates but higher Lysholm knee function scores than those managed with a traction table^8^. However, the effect of DRTR application on unstable intertrochanteric fractures remains elusive. In this study, we compared the outcomes of displaced intertrochanteric fractures treated with a DRTR or traction table to investigate the efficacy of DRTR in the management of unstable intertrochanteric fractures.

The aims of this study were: (i) to present a novel reduction strategy and to introduce a surgical maneuver for the management of unstable intertrochanteric fractures using a DRTR; (ii) to report the key skills required to manipulate the DRTR for unstable intertrochanteric fracture treatment; and (iii) to compare the clinical and radiological results of the two approaches in the treatment of unstable intertrochanteric fractures.

## Patients and Methods

### Inclusion and Exclusion Criteria

The inclusion criteria for this study were: (i) patients aged greater than 18 years with primary unilateral closed intertrochanteric fracture defined as AO/OTA classification type 31‐A2.2, 31‐A2.3, and 31‐A3 proximal femoral fractures; (ii) patients that have undergone closed reduction fixed with the PFNA‐II (Synthes, Oberdorf, Switzerland) facilitated with a DRTR or a traction table; and (iii) complete demographic and follow‐up data.

The exclusion criteria for this study were: (i) pathologic fractures and old fractures; (ii) patients combined with ipsilateral femoral neck fractures and femoral shaft fractures; (iii) open injuries; and (iv) patients with incomplete data during the follow‐up and less than 12 months of follow‐up.

The study protocol was reviewed and approved by the ethics board of the Second Xiangya Hospital of Central South University.

### Preoperative Examination and Treatment

After admission, the patients’ lower limb was provisionally fixed by skin traction. The latest guidelines of the American Academy of Orthopedic Surgeons recommend that elderly patients with intertrochanteric fractures receive surgery within 48 h after admission^4^. However, elderly patients often have medical comorbidities, such as chronic heart disease, type 2 diabetes mellitus, and pulmonary disease, which means that they are intolerant to surgery. Thus, it was important to evaluate the general condition of elderly patients before surgery was performed.

### Surgical Method

*Position and anesthesia*. The patients were placed in a supine position under general anesthesia on the radiolucent table. A folded compress was placed under the sacrum of the same side to eliminate the external rotation of the proximal fragment. The leg was disinfected with iodine complex from the ipsilateral costal margin to the ankle.

**Fig 1:**
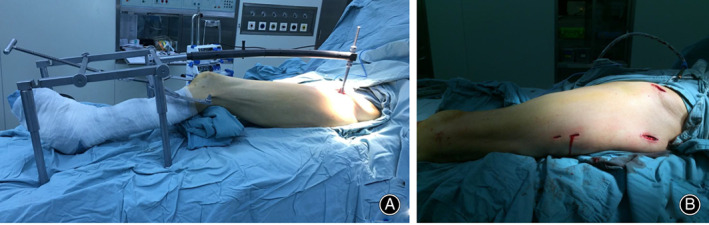
Intraoperative view of double reverse traction repositor application. (A) The double reverse traction repositor is connected to the anterior superior iliac spine (ASIS) *via* its proximal insertion and to the distal femur by traction bow at the distal end. (B) The minimally invasive incision for closed reduction of the intertrochanteric fracture and proximal femoral nail antirotation (PFNA‐II) insertion.

**Fig 2:**
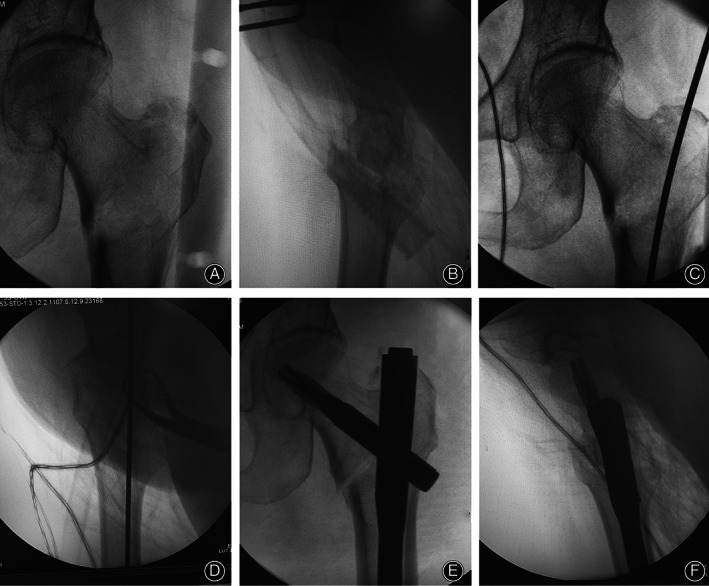
Intraoperative fluoroscopy images. Anteroposterior (A) and lateral (B) radiographic images of closed reduction facilitated by double reverse traction repositor. Quality of reduction is accepted as “excellent.” Anteroposterior (C) and lateral (D) radiographic images of the guide wire insertion. Anteroposterior (E) and lateral (F) radiographic images of the PFNA‐II nailing demonstrated that the anatomic reduction was maintained by the double reverse traction repositor and that the fixation quality was optimal.

*Exposure and approach*. When an acceptable fracture reduction was achieved, the exposure was performed. A 3‐cm proximal and longitudinal incision in line with the axis of the femur was made at the top of the greater trochanter through the fascia and gluteus to expose the entry point of the PFNA‐II (Fig. 1). Confirmed by C‐arm fluoroscopy, the guidewire was inserted into the lateral trochanteric apex. After reaming the proximal femoral canal, the PFNA‐II was inserted manually. The guidewire of the blade was positioned in the center or middle–lower third of the femoral neck in anteroposterior (AP) view and in the central or minor posterior of the femoral neck in lateral view (Fig. 2). If the location of the guidewire was unacceptable, adjustment of the guide pin position was needed through rotation, deeper insertion, or partial retraction of the PFNA‐II. The tip–apex distance (TAD) should be no more than 25 mm after insertion of the PFNA‐II blade. It was also noted that protrusion of the nail‐tail should be avoided.

**Fig 3:**
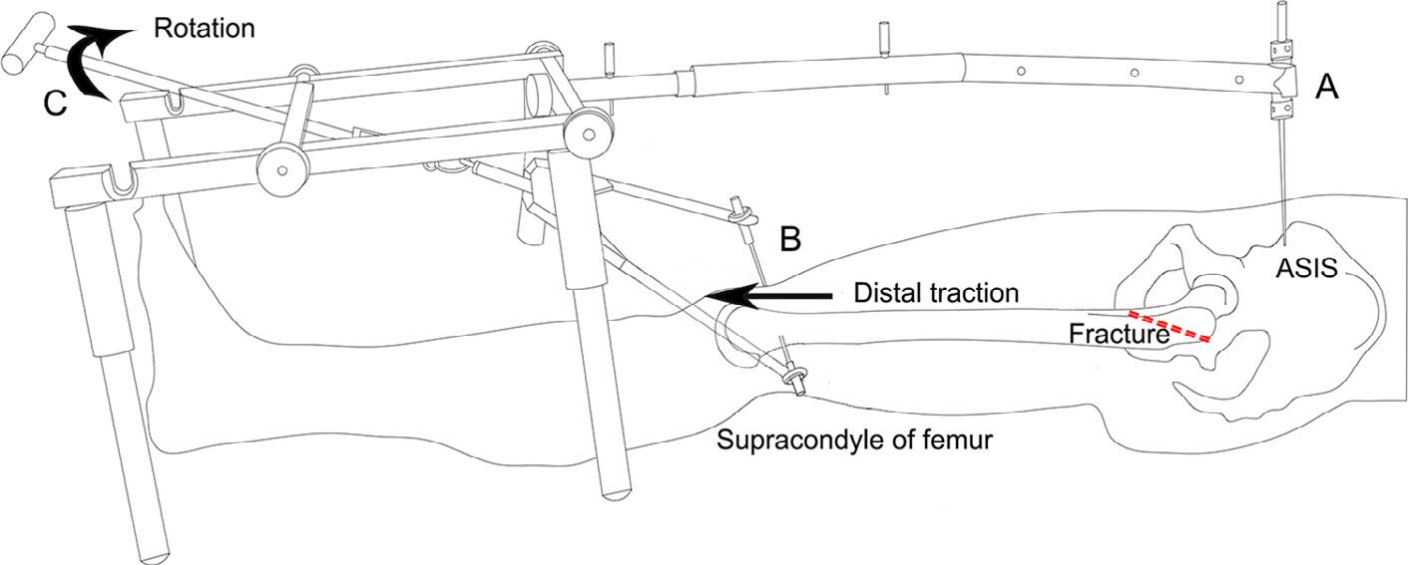
Schematic diagram of the double reverse traction repositor (DRTR) procedure: (A) an incision and drill of the anterior superior iliac spine (ASIS) was applied to screw in a 3‐mm Schanz pin. (B) A 2.5‐mm Kirschner wire was screwed in at the supracondyle of the femur to connect the traction bow. (C) After final installation of the DRTR, clockwise rotation of the handle of the reducer was performed to pull the distal femoral fracture fragment distally.

In the DRTR group, assembly of the DRTR on the surgical table was performed after the affected leg was prepared. A schematic illustration of the DRTR application is shown in Fig. 3. The repositor consists of a reduction scaffold, a traction bow, a traction pin, a radiolucent connecting rod, a distal reduction pin, and a proximal anchor (Figs 1 and 3). First, a 2‐cm incision was made and drilling of the ipsilateral anterior superior iliac spine (ASIS) was performed to screw in a 3‐mm Schanz pin (Figs 1A and 3A). The Schanz pin was linked to the proximal end of the radiolucent connecting rod *via* a cardan shaft. The optimal length of the connecting rod was selected according to the distance between the ASIS and the femoral condyle. Then, the distal end of the rod was fixed to the traction scaffold with four legs that can be adjusted to fit the height. Another 2.5‐mm Kirschner wire was screwed in at the supracondyle of the femur to connect the traction bow (Fig.3B). After that, the link between the traction bow and the rotary screw of the scaffold was created. Once firmly assembled, the intraoperative distraction and reduction were carried out by rotating the handle of the reducer clockwise to pull the distal femoral fracture fragment distally, under C‐arm fluoroscopy in anteroposterior and lateral views, thereby correcting the varus angulation and short deformity (Fig.3C). The external or internal rotation deformity could also be addressed by adjusting the traction bow inward or outward. For the lateral displacement, a Kirschner wire was inserted into the femur to assist the reduction. When acceptable fracture reduction was achieved, the standard process of PFNA‐II insertion was performed as described above.

In the traction table group, the closed reduction was performed according to the standard process. The affected side of the foot was firmly fixed in the boot while the contralateral leg was positioned flexed and abducted to allow easy access of C‐arm. An acceptable reduction was obtained with axial traction, slight internal rotation, and abduction of the fractured limb extremity. If the reduction was unacceptable, a minimal incision was made and a periosteal elevator or a Kirschner wire was applied to facilitate the reduction. When an acceptable reduction was confirmed in both AP and lateral views by image intensifier, the PFNA‐II was implanted in all patients (Fig.2). The optimal nail length and diameter were determined based on the femoral geometry of patients. Finally, static or dynamic distal locking was performed depending on the fracture stability after reduction. After the placement of the implant was confirmed by fluoroscopy, the tail cap was screwed on.

### Postoperative Rehabilitation

The isometric quadriceps exercise and an ankle pump exercise were performed on the first day after surgery. Active flexion and extension of the hip and knee were encouraged, and X‐rays were reviewed on postoperative day two. Low molecular weight heparin was prescribed for anticoagulation on the first day after surgery. Full weight‐bearing was permitted only when the fracture line on X‐rays and pain of the hip had disappeared.

### Patient Evaluation

The duration of the operation, the duration of patient positioning, and intraoperative blood loss were compared between the two groups. The operative blood loss was calculated using the Gross formula. The periods of hospitalization and postoperative complications were also reviewed. Fracture reduction quality and implant placement were assessed by two independent senior attending physicians. In cases of disagreement, another senior chief physician was consulted. During the follow‐up, the fracture healing time and partial weight‐bearing time were recorded. The HHS was evaluated at the last follow‐up.

#### Blood Loss

The Gross formula was applied to estimate the blood loss during surgery. Intraoperative actual blood loss = BV × (Hct_pre_ − Hct_pos_)/Hct_m_. Body volume (BV) = 70 mL × body weight (kg). Hct_pre_: Preoperative hematocrit. Hct_pos_: postoperative hematocrit. Hct_m_: mean perioperative hematocrit.

#### Fracture Reduction and Fixation Quality

The fracture reduction quality was evaluated based on Garden alignment index^9^. The fracture reduction was categorized as excellent, good, or poor. The criteria are as follows: (i) anatomic or slight valgus alignment in AP view, with no more than 20° of angulation in lateral view and less than 4 mm of displacement of any fragment considered to be excellent; (ii) a reduction that only meets the alignment or fragment displacement criteria in an excellent reduction is viewed as good; and (iii) a poor reduction was defined as neither alignment or fragment displacement criteria for an excellent reduction being met. To assess the fixation quality, the position of the blade within the femoral head and the TAD was analyzed^10^. Central–central and inferior–central placement of the blade as well as TAD < 25 mm both in AP and lateral view were recognized as optimal fixation. Suboptimal placement was defined as a placement that did not meet this criteria.

#### Harris Hip Score

The Harris hip score (HHS) was applied to measure the postoperative recovery of hip function. An HHS score <70 was categorized as poor, 70–80 as fair, 80–90 as good, and 90–100 as excellent.

### Statistical Analysis

The continuous variables were compared using the two‐sample Student *t*‐test. The *χ* ^2^‐test or Fisher's exact test were performed for the categorical variable (quality of reduction, quality of fixation, HHS, and postoperative complications) statistical analysis. Two‐tailed *P* < 0.05 was considered significantly different. SPSS22.0 (SPSS, Chicago, IL, USA) was used to perform the statistical analysis.

## Result

### General Results

From April 2015 and December 2018, a total of 95 patients met all the inclusion criteria. Among them, 56 patients were treated with DRTR and another 39 patients were treated with the traction table. In the DRTR group, 50 cases were diagnosed with 31A2 fractures and 6 patients were considered as having 31A1 fractures. The mean age of patients was 74.2 years. Of the 39 cases in the traction table group, 33 cases had 31A2 fractures and 9 patients had 31A3 fractures in the traction table group. The mean time from injury to surgery was comparable between the two groups (7.5 days for DRTR and 6.9 days for traction table, *P* = 0.22). General data for the enrolled cases is presented in Table 1. There were no significant differences regarding age, gender, fracture classification, and mechanism of injury between the two groups.

**TABLE 1:** Patient demographics and fracture characteristics

	DRTR group	TT group	*P*‐value
Gender (female/male)	34/22	30/9	0.10
Age (years)	74.2 ± 12.2	78.8 ± 10.3	0.06
Mechanism of injury			0.75
Simple fall at home	46	33	‐
Traffic accident	10	6	‐
AO fracture classification			0.10
31 A2	50	30	‐
31 A3	6	9	‐
Follow‐up (months)	19.1 (range, 10–31)	19.8 (range, 13–28)	0.46

*χ^2^*‐test.

DRTR, double reverse traction repositor; TT, table traction.

### Intraoperative Outcomes

As detailed in Table 2, the mean patient positioning time in the DRTR group was 6.5 ± 1.2 min, whereas it was 17.9 ± 7.0 min in the traction table group (*P* < 0.0001). In addition, the total surgical time was longer for the traction table group compared with the DRTR group (72.5 ± 6.1 min for traction table *vs* 63.0 ± 4.1 min for DRTR, *P* < 0.001). The amount of intraoperative blood loss was similar in both groups (68.9 ± 49.7 mL for DRTR and 154.1 ± 38.9 mL for traction table, *P* = 0.12).

**TABLE 2:** Comparison of surgical data and postoperative clinical outcome

	DRTR group	TT group	*P*‐value
Time from injury to surgery (days)	7.5 ± 2.3	6.9 ± 2.0	0.22
Duration of patient positioning (min)	6.5 ± 1.2	17.9 ± 7.3	<0.0001
Operative time (min)	63.0 ± 4.1	72.5 ± 6.1	<0.001
Intraoperative blood loss (mL)	168.9 ± 49.7	154.1 ± 38.9	0.12
Part loading time after surgery (days)			
A2	29.7 ± 4.8	28.8 ± 5.1	0.43
A3	44.0 ± 6.3	47.2 ± 6.9	0.70
Fracture healing time (weeks)	20.6 ± 2.3	21.4 ± 3.4	0.18
Harris hip score (cases)			0.98*
Excellent	10	8	‐
Good	36	24	‐
Fair	8	6	‐
Poor	2	1	‐

^*^ Fisher's exact test.

DRTR, double reverse traction repositor; TT, table traction.

### Outcome of Reduction and Fixation

For radiological assessment of the quality of the reduction and fixation, radiographs were taken on postoperative day 2. As shown in Table 3, in the DRTR group, excellent reductions were obtained in 39 patients (69.6%), good reductions in 14 patients (25.0%), and poor reductions in 3 patients (5.3%). In the traction table group excellent reductions were obtained in 19 patients (48.7%), good in 16 patients (41.0%), and poor in 4 patients (11.1%) The differences between the two groups were not statistically significant (*P* = 0.11). In the comparison of the fixation quality in reductions, the placement of blades was optimal in 80 patients (84.2%). There were no significant differences between the two groups in fracture reduction and implant position (*P* > 0.05).

**TABLE 3:** Details of early postoperative radiological evaluations for reduction and fixation quality

	DRTR group	TT group	*P*‐value
Quality of reduction			0.11*
Excellent	39	19	‐
Good	14	16	‐
Poor	3	4	‐
Quality of fixation			0.93^†^
Optimal	47	33	‐
Suboptimal	9	6	‐

^*^ Fisher's exact test.

^†^ χ^2^‐test.

DRTR, double reverse traction repositor; TT, table traction.

### Postoperative Follow‐Up

#### Follow‐Up Periods

The duration of follow up ranged from 12 to 31 months in total patients. The average follow up was 19.2 ± 4.7 months in the DRTR group and 19.8 ± 4.0 months in the traction table group, with no significant difference (*P* = 0.46).

#### Fracture Healing Time

The mean part loading time in 31A2 fractures was 29.7 ± 4.8 days for DRTR and 28.8 ± 5.1 days for the traction table, which did not reach statistically significant difference (*P* = 0.43). As for 31A3 fractures, the mean part loading time after surgery was 44.0 ± 6.3 days in the DRTR group and 47.2 ± 6.9 days in the traction table group without significant difference (*P* = 0.70). Both groups had similar mean fracture healing times (20.6 ± 2.3 weeks in the DRTR group and 21.4 ± 3.4 weeks in the traction table group).

#### Harris Hip Score

At the last follow‐up, the HHS was excellent in 10 patients (17.9%), good in 36 (64.3%), fair in 8 (14.3%), and poor in 2 (3.6%). These scores were comparable to those in the traction table group: excellent in 8 patients (20.5%), good in 24 (61.5%), fair in 6 (15.4%), and poor in 1 (2.6%) (*P* = 0.98, Fisher's exact test).

### Complications

As indicated in Table 4, an elderly patient with severe osteoporosis had an iatrogenic ASIS fracture and was then assigned to the tract table group. Because the avulsion fragment was too small to fix, it was removed. Postoperative complications occurred in 11 patients in the DRTR group and in 5 patients in the traction table group. No significant differences were found between the two groups in regard to postoperative complications (*P* = 0.83).

**Table 4:** Intraoperative and postoperative complications

	DRTR group	TT group	*P*‐value
Complications			0.83*
Thigh pain	6	3	‐
Deep vein thrombosis	4	2	‐
Fracture of ASIS	1	0	‐

^*^ Fisher's exact test.

ASIS, anterior superior iliac spine; DRTR, double reverse traction repositor; TT, table traction.

Thigh pain developed in 6 patients in the DRTR group and in 3 patients in the traction table group. After administration of nonsteroidal anti‐inflammatory drugs and physical therapy, these patients experienced significant improvement in thigh pain. In the DRTR group, 4 patients suffered from deep vein thrombosis, while this was observed in 2 patients in the traction table group. The deep vein thrombosis was not life threatening in any cases and was cured by anticoagulation treatment.

## Discussion

Dealing with an unstable intertrochanteric fracture is challenging for orthopaedic surgeons^11^. There is no consensus on the ideal therapeutic approach for unstable intertrochanteric fracture management. Both the choices of implant fixation and reduction quality are the foremost factors affecting the prognosis for unstable intertrochanteric fractures^12^. Recently, increasing evidence has suggested that cephalomedullary nailing is a suitable strategy due to its superior biomechanical and biological advantages relative to other approaches such as dynamic hip screw DHS and proximal femoral locking plates. Although the traction table is widely used for hip fracture closed reduction, it is associated with a variety of devastating complications. Emerging as a novel instrument for closed reduction of fractures, DRTR has been demonstrated to have superior efficacy in the management of femoral fractures^8^. Based on our knowledge, this is the largest study to compare the results of the DRTR and the traction table facilitating PFNA‐II fixation for the treatment of unstable intertrochanteric fractures.

We found that total operative time was shorter in the DRTR group compared with the traction table group. Compared to the traction table group, the decreased time required for patient positioning and easy abduction and adduction of the hip joint when inserting the guidewire may account for the shorter operative time in the DRTR group. In the traction table group, the mean duration of patient positioning and fracture reduction was comparable with that in a report by Wang *et al*.^13^. However, the mean duration of patient positioning in the DRTR group was only 6.5 ± 1.2 min, which was significantly shorter relative to the traction table group, possibly due to the easy assembling of the DRTR. Of note, the decreased operative time would substantially diminish the mortality rate in the surgical treatment of hip fractures, especially for the elderly who may be too fragile to tolerate a prolonged operation.

Recently, management of unstable intertrochanteric fractures using the PFNA‐II has arisen as a promising approach^14^. Growing evidence has demonstrated that excellent intertrochanteric fracture reduction is required before intramedullary nailing; otherwise, nail fixation failure is inevitable^15^. We discovered that satisfactory clinical efficacy was obtained when unstable intertrochanteric fractures were treated by double reverse traction with the PFNA‐II. The results showed that more than 80% of patients obtained excellent–good HHS at the end of follow‐up, which indicated that the application of DRTR would be an effective approach to improve postoperative hip function.

A high‐quality reduction is critical for attaining the ideal position of the PFNA‐II and achieving a good clinical outcome. In the DRTR group, postoperative X‐ray evaluation showed an excellent–good reduction in 95% of cases. Central–central and inferior–central placement of blades in the femoral neck were achieved in 47 cases (84%). These data indicated that application of DRTR was effective in assisting with unstable intertrochanteric fracture reduction. Generating pull force through direct skeletal traction, DRTR had obvious advantages over the traditional traction table. As the traction table generates force through pulling the distal extremity away from the perineum, the traction force need step over hip, keen and ankle joints before transmitting to the fracture site. This kind of skin traction would fail to provide sufficient force to correct the angular and rotational displacement. Therefore, a powerful and prolonged traction would be necessary, significantly increasing the risk of iatrogenic nerve injury and soft tissue damage^16^. In contrast, DRTR is fixed to the ASIS and distal femur using a traction bow; thereby, a skeletal traction system is formed. Once the distal tract handle was reverse‐rotated, consistent and sufficient force was generated to correct varus deformity and malalignment. As the resistant force by the ASIS *via* connection rod pulling can counter the distal femoral traction force, there is no need to place a perineal post, required for the traction table, which avoids the complications resulting from the compression of the labia or the scrotum^7^. Our data showed that no patients in the DRTR group suffered from peroneal nerve palsy, perineal ulcers, or nerve injury during the traction.

When performing intertrochanteric fracture reductions, techniques should be used to correct the varus angulation, external rotation, and posterior sag of the proximal fragment. Traditionally, table traction has been a popular procedure for intertrochanteric fracture reduction *via* longitudinal traction and internal rotation of the distal fragment. However, in certain intertrochanteric fractures, external rotation of the proximal fragment caused by short external rotators may compromise the reduction^17^. Riehl and Widmaier report that in a fracture with more than two independent fragments, especially a type A2 fracture with a posteromedial fragment, when internal rotation to the distal injury limb is applied, malunion and deformity can occur, and revision surgery is required^18^. In this regard, the rotation deformity could be effectively restored using a rotating traction bow in the DRTR group. Moreover, with the elevation of the injured limb by double reverse traction, the skeletal traction can not only correct the external rotation of the proximal fragment to some extent but also lift the level of entry point in the great trochanter to ease the PFNA‐II insertion.

The DRTR also allowed ipsilateral adduction or abduction to facilitate fluoroscopy and PFNA‐II nailing. The key step of the operation is locating the optimal entry point for PFNA‐II insertion. Generally, the best entry point is at the top of the greater trochanter or slightly medial^19^. Therefore, moderate adduction of the hip is required for surgeons to easily locate the top of the greater trochanter and insert the PFNA‐II, especially for obese patients. Compared to the traction table group, the fractured limb in the DRTR group could be easily placed in the desired position, which is of great help for PFNA‐II nailing. Taken together, the DRTR allows internal rotation of the hip for anatomic reduction as well as adduction for optimal insertion of the PFNA‐II, making it a promising instrument for treating unstable intertrochanteric fractures.

It must be noted that one elderly patient from the DTRD group had an iatrogenic ASIS fracture. When the DTRD is applied in the elderly, the bone mineral density should be measured to rule out severe osteoporosis, which may cause ASIS avulsion during the skeleton traction. Based on our experience, having more than 3 cm in distance between the drilling location and the surface of the ASIS and having a Schanz pin size of less than 3 mm would significantly decrease the risk of ASIS avulsion.

Although an extra incision in the ASIS and drilling in the ASIS and the femoral condyle were required in the DTRD group, no significant differences in mean blood loss was found between the two groups. The 2‐cm incision without needing further tissue and muscle dissection caused only a small amount of blood loss. Moreover, the cost of the double reverse device is less than 40,000 yuan (US$5000), which is much cheaper than the traction table, and is highly cost‐effective, especially for developing countries.

This study has several limitations. First, this report was retrospective and has inherent drawbacks. Without patients divided randomly, bias of the fracture reduction strategy selection was inevitable. An additional limitation was the small sample size and short follow‐up in our study. Therefore, a prospective study with more patients enrolled should be conducted to further investigate the application of DTRD for femoral intertrochanteric fracture treatment.

### Conclusion

The DRTR was determined to be a safe and effective approach to assist PFNA‐II treatment for unstable intertrochanteric fractures. Comparable results were achieved in the DRTR and traction table groups. However, the DRTR is superior to the tract table in respect to operative time and duration of patient positioning, despite the need for an additional ASIS incision and drilling of the ASIS and the femur condyle.
